# Chromosome 1p31.1 Deletion: A Case With Developmental Delay, Hypotonia, Cryptorchidism, Abnormal Oral Frenulum, and Feet Deformity

**DOI:** 10.1155/crig/6152118

**Published:** 2025-07-27

**Authors:** Tatiana Mikhailova, Ria Garg

**Affiliations:** ^1^Department of Psychiatry, State University of New York Upstate Medical University, Syracuse, New York, USA; ^2^Center for Development, Behavior, and Genetics, State University of New York Upstate Medical University, Syracuse, New York, USA; ^3^Department of Pediatrics, Upstate Golisano Children's Hospital, Syracuse, New York, USA

**Keywords:** case study, chromosome 1p31.1 deletion, cryptorchidism, de novo deletion, development, intellectual disability, microcephaly

## Abstract

Deletions within the chromosomal locus 1p31.1 are rare, with only a limited number of documented cases. The typical clinical presentation includes intellectual disability, failure to thrive, and craniofacial abnormalities. Some cases may also present with cardiac, gastrointestinal, and genitourinary malformations. Variability in deletion size contributes to a broad spectrum of clinical phenotypes, and a comprehensive understanding of the syndrome's manifestations is still evolving. This case study aims to provide additional insights into 1p31.1 microdeletion syndrome, enhancing knowledge of its genetic and phenotypic characteristics to improve recognition by clinicians. Here, we report a case featuring a 14.385 Mb deletion isolated to the 1p31.1 region, encompassing 41 genes. The deletion manifested with microcephaly, distinctive facial morphology, hypotonia, developmental delay, bilateral cryptorchidism, and flat feet. Notably, our case also exhibited congenital thickening of the lingual and labial frenulum, a trait not typically associated with this deletion.

## 1. Introduction

The chromosome 1p31.1 deletion is among the less described microdeletion syndromes, typically presenting with developmental delay, intellectual disability, craniofacial malformations, and other systemic abnormalities [1–3]. In 1979, Bene et al. reported the case of a 14 year-old girl with severe psychomotor delay, short stature, and dysmorphic features linked to a partial deletion of the short arm of Chromosome 1 [4]. Subsequent case reports have documented additional individuals with similar deletions, characterized by developmental delay and various congenital malformations [5–10]. Advances in genomic technology have enabled more precise localization and annotation of the affected genomic regions. Despite the phenotypic similarities across cases, understanding the genotype–phenotype relationship has been challenging due to variability in deletion size and breakpoints, which leads to involvement of different genes in each case [1, 3, 11–13].

This case study reports the first documented occurrence of a de novo deletion spanning 14.385 Mb, encompassing the genomic coordinates 70,438,241–84,822,837 on the p-arm of Chromosome 1. Alongside the established syndrome-associated traits such as microcephaly, hypotonia, and delays in psychomotor and speech development, we describe uncommon presentations of early postnatal bilateral cryptorchidism and thickening of the lingual and labial frenulum. These manifestations, while not emphasized in prior case studies, could be notable features of 1p31.1 deletion. We provide a comprehensive clinical overview of the case, contrasting the observed phenotypic profile with those documented in previous case studies with deletion coordinates mapped to the 1p31.1 locus.

## 2. Case Description

The patient described in this report is a 2-year-old male, delivered at full term to a 24-year-old mother (G2P0 > 1) and a 25-year-old nonconsanguineous father. The pregnancy was complicated by maternal hypertension but was otherwise unremarkable, with no infections, diabetes, medication use, or exposure to tobacco, alcohol, or drugs. Fetal ultrasounds showed normal development and movements. Delivery was complicated by a cesarean section due to failed induction. Birth measurements were as follows: length 50.8 cm (23rd percentile), weight 2667 g (7th percentile), and head circumference 32 cm (5th percentile), with Apgar scores of 8 and 9 at 1 and 5 min, respectively. No abnormalities were noted at birth.

On the third day after birth, the patient presented to the emergency department with breathing difficulties following milk aspiration. Evaluation revealed hypothermia, hypoglycemia, and respiratory distress, necessitating a 5-day stay in the neonatal intensive care unit (NICU). In the NICU, ankyloglossia and a tight upper labial frenulum were noted, prompting lingual and upper labial frenulectomies. Partial recurrence of the banding was observed at a 3-month follow-up, though no repeat procedure was recommended due to lack of functional impairment.

At the 2-month pediatric evaluation, the patient's development was reported as normal, with no concerns identified. At 4 months, however, hypotonia and motor developmental delays were observed, prompting a referral to the child neurology clinic. Evaluation by the neurologist revealed generalized hypotonia, difficulty with head and chest lifting, lack of coordination, poor weight gain, and borderline microcephaly (−2 SD). His measurements were as follows: height 64.3 cm (47th percentile), weight 5.05 kg (< 1st percentile), and head circumference 38.8 cm (< 1st percentile). Physical examination showed dysmorphic features, including a long face with a narrow and tall forehead, closely spaced eyes, a high-arched narrow palate, a broad nasal tip, and micrognathia. Given these findings, a genetic etiology was suspected, and chromosomal microarray testing was ordered.

At 10 months, the patient was evaluated by pediatric urology for bilateral undescended testicles, requiring bilateral orchidopexy. An ophthalmology assessment at that time revealed hyperopia. At 19 months, an ear, nose, and throat (ENT) examination found malalignment of mandibular incisors, likely due to the initial ankyloglossia, contributing to dental malocclusion. A musculoskeletal evaluation at age 2 by the orthopedic surgery department identified bilateral flat feet and partial cutaneous syndactyly between the second and third toes, characterized by the second toe overlapping the third. The developmental history indicated delays in motor milestones, with head control achieved at 5 months, independent sitting at 7 months, crawling at 9 months, and walking at 18 months.

**Figure 1:**
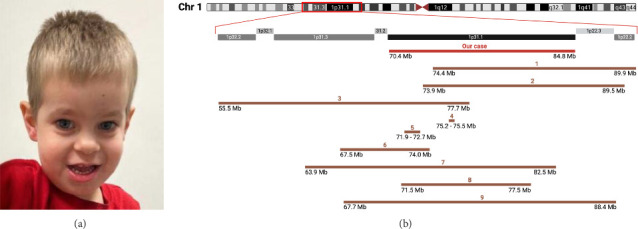
(a) Facial characteristic noted at 2 years old: long face, tall forehead, closely spaced eyes, broad nasal tip, prominent low-set ears, micrognathia, and tendency for an open-mouth posture. (b) Comparison of 1p31.1 70,438,241–84,822,837 deletion interval with previously reported cases (Cases 1–9, Table 1).

Genetic screening results were delayed due to a transfer of care between hospitals. At 2 years, chromosomal microarray analysis revealed a pathogenic 14.385 Mb deletion on chromosome 1p31.1 (70,438,241–84,822,837), involving 41 protein-coding genes. On follow-up evaluation at the pediatric genetics department, previously documented dysmorphic features and an open-mouth posture were noted (Figure 1(a)). Language delay was noted, with the patient speaking his first words at 12 months. During evaluation, he had a limited vocabulary and was unable to form short sentences. He also displayed episodic impulsivity and an abnormal gait. Growth parameters showed head circumference below the 5th percentile, with height and weight within normal ranges.

Given the limited knowledge of the syndrome, management recommendations focused on addressing symptomatic concerns through a multidisciplinary approach. A renal ultrasound and cardiological evaluation, including an EKG and echocardiogram, were performed, all yielding unremarkable findings. At a neurological evaluation at 2 years 11 months, the patient's height and weight were in the 30th and 23rd percentiles, respectively, with a head circumference below the 2nd percentile. He was described as friendly, enjoyed interacting with others, showed no repetitive behaviors or stereotyped movements, maintained good eye contact, and exhibited no sensory issues. However, delayed speech development was noted; his vocabulary consisted of 20–30 words, and he primarily communicated in 1–2 word phrases, with echolalia also observed. Physical examination revealed diffusely low muscle tone and hypermobile joints. While he exhibited no difficulties with walking or running, he demonstrated poor balance and coordination. To address these challenges, the patient was enrolled in early intervention programs, including speech, physical, and occupational therapy.

## 3. Discussion

The individual presented in this case report has a notable deletion within the chromosome 1p31.1 locus, encompassing 41 genes, with the distal breakpoint located within the *LRRC7* coding region (Supporting Table 1). Hallmark features of 1p31.1 deletion include intellectual disability, delayed psychomotor development, speech articulation difficulties, hypotonia, growth delay, and dysmorphic facial features such as a prominent forehead, microcephaly, micrognathia, broad nasal tip, and epicanthal folds (Figure 1(a) and Table 1). Similar clinical characteristics, including genitourinary anomalies and foot abnormalities, have been observed in cases from the DECIPHER database (Table 2). Although inherited cases of the deletion have been documented [12, 16], the majority arise from de novo mutations [2, 4], sometimes due to genetic translocation events in healthy parents [13]. In our case, parental testing for the deletion yielded normal results, confirming its de novo origin. Testing for potential balanced rearrangements in parental samples was not conducted.

The low incidence of 1p31.1 deletion, variability in the deleted genomic intervals, and potential pleiotropic effects obscure understanding of how the absence of specific genes contributes to the observed phenotype. However, it is apparent that the severity of symptoms exhibits a dose-dependent effect, with more extensive deletions associated with more pronounced clinical presentations (Table 1). Larger deletions of approximately 20 Mb within 1p32.2–1p22.1 loci resulted in severe craniofacial abnormalities and congenital malformations that impact fetal viability (Figure 1(b) and Table 1) [11–13]. Intermediate deletions within the 15 Mb range were not discernible during prenatal and early postnatal evaluations. Affected individuals manifested symptoms around 4–6 months of age, characterized by psychomotor delay evident as an inability to support the head. This pattern was observed in our case, as well as in a previously described report involving a 15.5 Mb deletion spanning from 1p31.1 to 1p22.2 [14]. The deletion of a single gene or a short noncoding sequence may result in phenotypes insufficiently apparent to suggest a genetic etiology during early childhood [3, 16]. For example, an individual with a single gene deletion did not undergo evaluation for genetic defects until concerns regarding learning impairment emerged at 7 years of age, even when impulsivity, hyperactivity, and speech delays were noted at 13 months [3]. A smaller microdeletion of 0.27 Mb lacking protein-coding genes did not result in any abnormalities except thickened labial frenulum and dental misalignment [16]. Frenulum abnormalities were also observed in our case, requiring surgical intervention, as tight frenula in newborns can impair tongue mobility, interfere with breastfeeding, and contribute to early nutritional deficiencies [17].

Cognitive delay and neurological manifestations, including speech and psychomotor disturbances, as well as attention and memory deficits, are the most common manifestations of 1p31.1 deletion (Table 1). Rivera-Pedroza et al. provided detailed insight into a case featuring a 18.6 Mb 1p31.1 through p31.3 deletion, which presented severe physical and developmental challenges [12]. In addition, Biswal et al. described a case involving an 11-year-old male who had a 5.99 Mb deletion at 1p31.1, which included 18 genes that are also absent in our case. The individual described by the authors demonstrated moderate intellectual disability, inattention, hyperactivity, and severe language impairment, with the emergence of first words only at age 5 and the ability to form short sentences achieved only at age 11 [1]. These difficulties mirror those observed in our case and other previous reports (Table 1). The study by Callier et al. provides insight into the long-term developmental trajectory of individuals with 1p31.1 deletion, describing a 20-year-old male with a 15.6 Mb deletion who exhibited moderate intellectual disability and remained unable to write, read, or count [15].

The observed neurological manifestations are likely attributable to a multifaceted interplay among numerous genes. The neuronal growth regulator *(NEGR1*), located at 71,861,626–72,748,222 on Chromosome 1 (Supporting Table 1), is frequently observed within the deleted intervals and has been previously described in the context of 1p31.1 deletion [3]. All but one of the documented cases in our study exhibit a *NEGR1* deletion, consistently presenting with psychomotor and developmental delays (Figure 1(b) and Table 1). Genovese et al. reported a case of two siblings with a deletion in the 71,868,625–72,748,533 region, affecting only the *NEGR1* gene, presenting with developmental and language delays, psychomotor retardation, and hypotonia [3]. The mechanism by which *NEGR1* deletion could contribute to these phenotypes is not fully understood due to limited human evidence. Mouse models have shown that *NEGR1* deficiency disrupts neurite outgrowth during neuritogenesis and alters the excitatory/inhibitory neuronal balance, resulting in social behavior impairments [18]. The NEGR1 protein has also been shown to interact with fibroblast growth factor receptor 2 (FGFR2), influencing cortical development via the ERK and AKT signaling pathways [19].

The distal breakpoint of the deletion we report falls within the *LRRC7* gene, resulting in the loss of 19 out of 25 exons. *LRRC7* encodes densin-180, a protein involved in maintaining postsynaptic neuronal signaling through interactions with signaling proteins, such as the α-subunit of Ca2+/calmodulin-dependent protein kinase II (αCaMKII) [20]. Truncations of the *LRRC7* gene result in a loss of protein function, as its C-terminal residues are essential for binding to αCaMKII, which modulates phosphorylation of synaptic proteins during synaptic plasticity. Individuals with loss-of-function variants in *LRRC7* consistently exhibit a phenotype characterized by intellectual disability, attention deficits, and global neurodevelopmental delay, including delays in motor and speech development [20].

Although cryptorchidism has rarely been reported as a characteristic of 1p31.1 deletion syndrome, it has been previously described by Callier et al. [15] and documented in the DECIPHER database in individuals with a similar deletion region (Tables 1 and 2). While the etiology of cryptorchidism remains unclear, the 1p31.1 deletion could plausibly disrupt hypothalamic–pituitary signaling, supported by a documented case of congenital hypopituitarism associated with 1p31.1 deletion [9]. Considering the potential consequences of failed testicular descent, such as an increased risk of testicular neoplasms and torsion, it is important to recognize cryptorchidism as a possible component of the syndrome and ensure timely management [21, 22].

The absence of a standardized management protocol for 1p31.1 deletion syndrome necessitates individualized treatment plans. Comprehensive developmental and neuropsychological assessments are essential, including speech therapy and evaluations of neurological, visual, and auditory systems. Palate defects, genitourinary abnormalities, abnormal muscle tone, growth restriction, and low-set ears occur in over 50% of cases and should be routinely assessed during clinical follow-up. Less common but clinically significant findings, such as cardiac anomalies, neuroimaging abnormalities, musculoskeletal issues (e.g., scoliosis, joint laxity, and toe deformities), seizures, and dental anomalies, should be evaluated and monitored based on clinical judgment. Among facial dysmorphic features, low-set ears, micrognathia, and epicanthal folds are present in over half of affected individuals, supporting their use as key features in diagnostic evaluation and dysmorphology assessments (Table 3). The variability of other clinical manifestations further underscores the need for personalized management strategies (Figure 1(a) and Table 1).

In conclusion, we report a case of a 14.385 Mb deletion in the 1p31.1 region which, in addition to developmental challenges including hypotonia and language delays, also presented with cryptorchidism and thickened frenula. These findings expand the phenotypic spectrum of 1p31.1 deletion syndrome by adding features not previously reported. We aim to contribute to the knowledge of this rare deletion to improve the diagnosis and management of affected patients, emphasizing their need for multidisciplinary care.

## Figures and Tables

**Table 1:** Summary of clinically observed abnormalities associated with 1p31.1 deletions reported in previous case studies.

	Our case	1	2	3	4	5	6	7	8	9	Total
		Maegawa et al. [14]	Callier et al. [15]	Chen et al. [11]	Yildirim et al. [16]	Genovese et al. [3]	Tassano et al. [2]	Rivera-Pedroza et al. [12]	Biswal et al. [1]	Serra et al. [13]	
Deleted region (Mb)	70.4–84.8	74.4–89.9	73.9–89.5	55.5–77.7	75.2–75.5	71.9–72.7	67.5–74.0	63.9–82.5	71.5–77.5	67.7–88.4	
Deletion length (Mb)	14.3	15.5	15.6	22.2	0.3	0.9	6	18.6	6	20.7	
Protein-coding genes (N)	41	71	65	84	0	1	19	69	18	76	
Age/sex	2 years/M	6 years/F	20 years/M	Nonviable 33 weeks/F	2–20 years/M/F siblings	12 years/M/F siblings	6 years/M	27 days/F	11 years/M	4 months/F	
Developmental delay/intellectual disability	+	+	+	NA	−	+	+	NA	+	+	7/8
Psychomotor delay	+	+	+	NA	−	+	+	NA	+	+	7/8
Language delay	+	+	+	NA	−	+	+	NA	+	+	7/8
Growth restriction	+	+	+	NA	−	+	+	NA	−	+	6/8
Impulsivity/attention deficit	+	+	−	NA	−	+	+	NA	+	NA	5/7
Abnormal muscle tone	+ Hypotonia	+ Hypotonia	−	NA	−	+ Hypotonia	+ Paratonia	NA	−	+ Hypotonia	5/8
											
Facial shape	+ Elongated, prominent forehead, micrognathia	+ Elongated, prominent forehead, micrognathia	+ Elongated, micrognathia	+ Elongated, prominent forehead, micrognathia	−	−	−	+ Micrognathia	−	+ Micrognathia	6/10
Periocular	+ Hypotelorism, epicanthal folds	+ Hypoplastic supraorbital ridges	+ Downslanting palpebral fissures, epibulbar dermoid cyst	+ Hypotelorism	−	+ Hypotelorism, epicanthal folds, narrow palpebral fissures	−	+ Hypotelorism, exophthalmos, absence of eyelids	−	+ Hypotelorism, microphthalmia, epicanthal folds	7/10
Auricular	+ Low-set	+ Low-set, prominent antihelix	+ Narrow	+ Low-set	−	+ Low-set, cupped-shaped	−	+ Low-set	−	+ Low-set, cupped-shaped	6/10
Nasal	+ Broad tip	+ Depressed nasal bridge	+ Elongated	+ Broad tip	−	−	−	−	−	+ Broad tip	4/10
Head circumference abnormality	+ Microcephaly	−	+ Microcephaly	+ Macrocephaly	−	−	+ Microcephaly	−	−	+	5/10
Neuroimaging abnormalities	−	+ Ventriculomegaly, focal intracerebral infarct	−	+ Hydrocephalus, ventriculomegaly, corpus callosum hypogenesis	NA	NA	NA	+ Hydrocephalus, ventriculomegaly, focal intracerebral hemorrhage craniosynostosis	−	+ Ventriculomegaly, corpus callosum agenesis, craniosynostosis	4/7
Palate abnormality/cleft lip	+ High-arched palate	+ High-arched palate	+ High-arched palate	−	+ Cleft lip	−	−	+ Cleft palate	−	+ Cleft palate	6/10
Dental	+ Poor alignment	−	+ Hypodontia, double dentition	NA	+ Poor alignment, diastemia	+ Enamel dysplasia	−	NA	−	NA	3/7
Thickened frenula	+	−	−	−	+	−	−	−	−	−	2/9
Tendency to open the mouth	+	+	+	NA	−	−	−	NA	−	NA	3/7
Visual impairment and oculomotor abnormalities	+ Hyperopia	−	+ Hypermetropia	NA	−	+ Hyperopia, strabismus	+ Strabismus	+ Ectopia lentis, sclerocornea	−	+ Strabismus, coloboma of iris/optic nerve	6/9
Hearing loss	−	−	+ Conductive	NA	−	−	−	−	−	+ Sensorineural	2/9
MSK	+ Syndactyly (2^nd^ to 3^rd^ toe), pes planus, joint laxity	+ Pectus excavatum	+ Incomplete extension of some fingers, clinodactyly (4th and 5th fingers), scoliosis	NA	−	+ Syndactyly (2^nd^ to 3^rd^ toe), joint laxity scoliosis	−	−	−	+ Pectus excavatum, calcaneovalgus talipes, crowded toes, adducted thumbs	5/9
Cardiac	−	+ VSD, PFO, PVS	−	−	−	+ AoR dilatation, MVP	−	−	−	+ PHTN, PFO	3/9
Genitourinary	+ Cryptorchidism	−	+ Cryptorchidism	+ Ambiguous external genitalia	−	+ Duplicated ureters, shawl scrotum	−	+ Renal hypoplasia	−	−	5/10
GI/metabolic	−	+ MCADD	−	NA	−	+ Hypothyroidism, projectile vomiting	−	−	−	+ Hepatic left segment hypertrophy, dysmorphic gallbladder	3/9
Skin	−	+ Inverted nipples	+ Psoriasis, palmar creases	NA	−	+ Hypopigmentation, cutis laxa	−	+ Cutis laxa	−	−	4/9
Neonatal RD	+	−	−	NA	−	−	−	−	+	+	3/9
Seizures	−	+	−	NA	−	−	−	−	−	−	1/9

*Note:* NA, not applicable due to developmental stage or phenotype not assessed; MSK, musculoskeletal; AoR, aortic root; PHTN, pulmonary hypertension; MCADD, medium-chain Acyl-CoA.

Abbreviations: GI, gastrointestinal; MVP, mitral valve prolapse; PFO,  patent foramen ovale; PVS, pulmonary valve stenosis; RD, respiratory distress; VSD, ventricular septal defect.

**Table 2:** Clinical characteristics of 1p31.1 10–19 Mb deletion cases reported in the DECIPHER database.

DECIPHER ID	248,394	266,358	359,769	526,048
Chr 1 deletion	69,352,774–87,931,338	71,592,911–91,190,903	66,521,179–80,724,081	68,227,446–79,115,231
Deletion region (Mb)	18.58	19.6	14.2	10.89
Clinical features	**Abnormal toe morphology, abnormality of the mouth, cryptorchidism,** downslanted palpebral fissures, facial asymmetry, gynecomastia**, high palate, hypotonia,** inguinal hernia, **intellectual disability, joint laxity**, microtia, pectus excavatum, preauricular skin tag, prominent fingertip pads, scoliosis, talipes equinovalgus	Anal atresia, hydronephrosis, **intellectual disability**	**Axial hypotonia,** broad forehead, **low-set ears,** overfolded helix, seizure, thickened helices	**Abnormality of the dentition, atypical behavior, bulbous nose,** convulsive status epilepticus, **high palate, hypotonia, intellectual disability, low-set ears, short chin, short nose**

*Note:* Clinical features observed in our case are in bold.

**Table 3:** Surveillance and management recommendations for individuals with 1p31.1 deletions.

Specialty areas	Clinical recommendations
Pediatrics/nutrition	Regular monitoring of growth parameters and nutritional support, given a 75% incidence of growth restriction
Developmental therapy	Regular neurodevelopmental evaluations and interventions, including physical, occupational, and speech therapy
Cardiology	Baseline cardiac evaluation (echocardiogram and cardiology consultation) as part of the initial clinical workup
Ophthalmology and audiology	Regular ophthalmology and audiology assessments
Urology	Routine screening for anomalies such as cryptorchidism
Neurology	Periodic evaluations, including neuroimaging were clinically indicated (67% prevalence of neuroimaging abnormalities) and EEG for seizures (rare)
Dentistry	Regular dental assessments
Orthopedics/physical medicine	Regular assessment for musculoskeletal anomalies, including scoliosis, joint laxity, and foot deformities
Genetic counseling	Families should receive genetic counseling for recurrence risk assessment and psychosocial support
Other	Gastroenterology, endocrinology, and dermatology referrals should be considered when metabolic or dermatologic abnormalities are suspected

## Data Availability

This study did not generate any new data.

## Supporting Information

Supporting Table 1: Protein-coding genes located within the Chr1: 70,438,241–84,822,837 deletion interval (GRCh37/hg19). The table provides functional context for the deleted genes and their potential relevance to the observed phenotype. Each gene is annotated with its NCBI ID, OMIM entry, chromosomal position, and associated Gene Ontology (GO) biological processes.

Supporting Table 2: Comparison of protein-coding genes deleted in our case with previously reported 1p31.1 deletion cases that share overlapping genomic coordinates. Overlapping genes are indicated in bold to highlight shared molecular disruptions across cases.
