# Supplementary material for: Chromosome 1p31.1 Deletion: A Case With Developmental Delay, Hypotonia, Cryptorchidism, Abnormal Oral Frenulum, and Feet Deformity
Source: Case Rep Genet. 2025 Jul 27;2025:6152118. doi: 10.1155/crig/6152118 (PMC12318624; doi:10.1155/crig/6152118)
Supplement: Supporting Information — Additional supporting information can be found online in the Supporting Information section. [file 6152118.f1.pdf]

Supplementary Table 1. Protein coding genes located within Chr1: 70,438,241–84,822,837 deletion interval in our patient (GRCh37/hg19).

	Gene	Name	NCBI	OMIM	Chr1 annotation	Gene Ontology (GO) – Biological Process [23]
1	*LRRC7*	Leucine Rich Repeat Containing 7	57554	614453	70,033,605–70,610,047	GO:0009790 embryo development<br>GO:0010976 positive regulation of neuron projection development<br>GO:0043113 receptor clustering<br>GO:0045197 establishment or maintenance of epithelial cell apical/basal polarity<br>GO:0098609 cell-cell adhesion
2	*LRRC40*	Leucine Rich Repeat Containing 40	55631	–	70,610,488–70,671,262	GO:0007165 signal transduction
3	*SRSF11*	Serine And Arginine Rich Splicing Factor 11	9295	602010	70,671,379–70,718,735	GO:0006397 mRNA processing<br>GO:0008380 RNA splicing
4	*ANKRD13C*	Ankyrin Repeat Domain 13C	81573	615125	70,724,682–70,820,405	GO:0006621 protein retention in ER lumen<br>GO:0010469 regulation of signaling receptor activity<br>GO:2000209 regulation of anoikis
5	*CTH*	Cystathionine Gamma-Lyase	1491	607657	70,876,951–70,905,534	GO:0006534 cysteine metabolic process<br>GO:0006629 lipid metabolic process<br>GO:0008652 amino acid biosynthetic process<br>GO:0018272 protein-pyridoxal-5-phosphate linkage via peptidyl-N6-pyridoxal phosphate-L-lysine<br>GO:0019343 cysteine biosynthetic process via cystathionine
6	*PTGER3*	Prostaglandin E Receptor 3	5733	176806	71,318,04–71,513,499	GO:0006954 inflammatory response<br>GO:0007165 signal transduction<br>GO:0007186 G protein-coupled receptor signaling pathway<br>GO:0007189 adenylate cyclase-activating G protein-coupled receptor signaling pathway<br>GO:0007200 phospholipase C-activating G protein-coupled receptor signaling pathway
7	*ZRANB2*	Zinc Finger RANBP2-Type Containing 2	9406	604347	71,528,974–71,546,718	GO:0006396 RNA processing<br>GO:0006397 mRNA processing<br>GO:0008380 RNA splicing
8	*NEGR1*	Neuronal Growth Regulator 1	257194	613173	71,861,626–72,748,222	GO:0007155 cell adhesion<br>GO:0007626 locomotory behavior<br>GO:0007631 feeding behavior<br>GO:0010976 positive regulation of neuron projection development<br>GO:0031175 neuron projection development

9	*LRRIQ3*	Leucine Rich Repeats And IQ Motif Containing 3	127255	617957	74,491,699–74,663,860	None
10	*FPGT*	Fucose-1-Phosphate Guanylyltransferase	8790	603609	74,663,926–74,674,386	GO:0006004 fucose metabolic process<br>GO:0042354 L-fucose metabolic process<br>GO:0044238 primary metabolic process<br>GO:0071704 organic substance metabolic process
11	*FPGT-TNNI3K*	FPGT-TNNI3K Readthrough	100526835	–	74,663,926–75,010,112	GO:0006468 protein phosphorylation<br>GO:0044238 primary metabolic process<br>GO:0071704 organic substance metabolic process
12	*TNNI3K*	TNNI3 Interacting Kinase	51086	613932	74,701,071–75,010,112	GO:0002027 regulation of heart rate<br>GO:0006468 protein phosphorylation<br>GO:0016310 phosphorylation<br>GO:0055117 regulation of cardiac muscle contraction<br>GO:0086069 bundle of His cell to Purkinje myocyte communication
13	*LRRC53*	Leucine Rich Repeat Containing 53	105378803	–	74,935,060–74,978,295	None
14	*ERICH3*	Glutamate Rich 3	127254	–	75,033,807–75,139,476	GO:0060271 cilium assembly
15	*CRYZ*	Crystallin Zeta	1429	123691	75,171,170–75,198,734	GO:0007601 visual perception<br>GO:0008152 metabolic process<br>GO:0042178 xenobiotic catabolic process<br>GO:0051289 protein homotetramerization
16	*TYW3*	TRNA-YW Synthesizing Protein 3 Homolog	127253	611245	75,198,836–75,232,361	GO:0008033 tRNA processing<br>GO:0030488 tRNA methylation<br>GO:0031591 wybutosine biosynthetic process<br>GO:0032259 methylation
17	*LHX8*	LIM Homeobox 8	431707	604425	75,594,119–75,627,218	GO:0006355 regulation of DNA-templated transcription<br>GO:0006357 regulation of transcription by RNA polymerase II<br>GO:0007611 learning or memory<br>GO:0008585 female gonad development<br>GO:0021879 forebrain neuron differentiation
18	*SLC44A5*	Solute Carrier Family 44 Member 5	204962	620329	75,667,814–76,081,848	GO:0006656 phosphatidylcholine biosynthetic process<br>GO:0015871 choline transport<br>GO:0055085 transmembrane transport
19	*ACADM*	Acyl-CoA Dehydrogenase Medium Chain	34	607008	76,190,394–76,229,364	GO:0001889 liver development<br>GO:0005978 glycogen biosynthetic process<br>GO:0006082 organic acid metabolic process<br>GO:0006111 regulation of gluconeogenesis<br>GO:0006629 lipid metabolic process

20	*RABGGTB*	Rab Geranylgeranyltransferase Subunit Beta	5876	179080	76,251,879–76,260,775	GO:0007601 visual perception<br>GO:0018344 protein geranylgeranylation<br>GO:0036211 protein modification process
21	*MSH4*	MutS Homolog 4	4438	602105	76,262,567–76,378,927	GO:0001541 ovarian follicle development<br>GO:0006298 mismatch repair<br>GO:0007129 homologous chromosome pairing at meiosis<br>GO:0007131 reciprocal meiotic recombination<br>GO:0007283 spermatogenesis
22	*ASB17*	Ankyrin Repeat And SOCS Box Containing 17	127247	619936	76,384,558–76,398,089	GO:0016567 protein ubiquitination<br>GO:0035556 intracellular signal transduction
23	*ST6GALNAC3*	ST6 N-Acetylgalactosaminide Alpha-2,6-Sialyltransferase 3	256435	610133	76,540,431–77,103,024	GO:0001574 ganglioside biosynthetic process<br>GO:0006486 protein glycosylation<br>GO:0006629 lipid metabolic process<br>GO:0006677 glycosylceramide metabolic process<br>GO:0006687 glycosphingolipid metabolic process
24	*ST6GALNAC5*	ST6 N-Acetylgalactosaminide Alpha-2,6-Sialyltransferase 5	81849	610134	77,333,165–77,533,231	GO:0001574 ganglioside biosynthetic process<br>GO:0006486 protein glycosylation<br>GO:0006629 lipid metabolic process<br>GO:0006688 glycosphingolipid biosynthetic process<br>GO:0009311 oligosaccharide metabolic process
25	*PIGK*	Phosphatidylinositol Glycan Anchor Biosynthesis Class K	10026	605087	77,554,674–77,685,115	GO:0006506 GPI anchor biosynthetic process<br>GO:0006508 proteolysis<br>GO:0016255 attachment of GPI anchor to protein
26	*AK5*	Adenylate Kinase 5	26289	608009	77,747,704–78,025,651	GO:0006139 nucleobase-containing compound metabolic process<br>GO:0006172 ADP biosynthetic process<br>GO:0006173 dADP biosynthetic process<br>GO:0009117 nucleotide metabolic process<br>GO:0009123 nucleoside monophosphate metabolic process
27	*MIGA1*	Mitoguardin 1	374986	616773	77,779,624–77,879,540	GO:0008053 mitochondrial fusion
28	*FUBP1*	Far Upstream Element Binding Protein 1	8880	603444	77,944,055–77,979,501	GO:0006355 regulation of DNA-templated transcription<br>GO:0010628 positive regulation of gene expression
29	*DNAJB4*	DnaJ Heat Shock Protein Family (Hsp40) Member B4	11080	611327	77,979,175–78,017,964	GO:0000122 negative regulation of transcription by RNA polymerase II<br>GO:0006457 protein folding<br>GO:0006986 response to unfolded protein<br>GO:0009408 response to heat<br>GO:0051085 chaperone cofactor-dependent protein refolding
30	*GIPC2*	GIPC PDZ Domain Containing Family Member 2	54810	619089	77,979,542–78,138,444	GO:0008150 biological_process

31	*ZZZ3*	Zinc Finger ZZ-Type Containing 3	26009	619892	78,028,101–78,149,083	GO:0006325 chromatin organization<br>GO:0006355 regulation of DNA-templated transcription<br>GO:0006357 regulation of transcription by RNA polymerase II<br>GO:0031063 regulation of histone deacetylation<br>GO:0044154 histone H3-K14 acetylation
32	*USP33*	Ubiquitin Specific Peptidase 33	23032	615146	78,161,672–78,225,537	GO:0006508 proteolysis<br>GO:0006511 ubiquitin-dependent protein catabolic process<br>GO:0006897 endocytosis<br>GO:0007411 axon guidance<br>GO:0008277 regulation of G protein-coupled receptor signaling pathway
33	*NEXN*	Nexilin F-Actin Binding Protein	91624	613121	78,354,309–78,409,580	GO:0007156 homophilic cell adhesion via plasma membrane adhesion molecules<br>GO:0007411 axon guidance<br>GO:0030334 regulation of cell migration<br>GO:0051493 regulation of cytoskeleton organization<br>GO:0070593 dendrite self-avoidance
34	*PTGFR*	Prostaglandin F Receptor	5737	600563	78,956,659–79,006,386	GO:0006954 inflammatory response signal transduction<br>GO:0007165 signal transduction<br>GO:0007186 G protein-coupled receptor signaling pathway<br>GO:0007189 adenylate cyclase-activating G protein-coupled receptor signaling pathway<br>GO:0007204 positive regulation of cytosolic calcium ion concentration
35	*IFI44L*	Interferon Induced Protein 44 Like	10964	613975	79,086,133–79,111,830	GO:0006955 immune response<br>GO:0051607 defense response to virus
36	*IFI44*	Interferon Induced Protein 44	10561	610468	79,115,474–79,129,763	GO:0006955 immune response<br>GO:0009615 response to virus<br>GO:0009617 response to bacterium
37	*ADGRL4*	Adhesion G Protein-Coupled Receptor L4	64123	616419	79,355,449–79,472,415	GO:0007165 signal transduction<br>GO:0007166 cell surface receptor signaling pathway<br>GO:0007186 protein-coupled receptor signaling pathway<br>GO:0007189 adenylate cyclase-activating G protein-coupled receptor signaling pathway
38	*ADGRL2*	Adhesion G Protein-Coupled Receptor L2	23266	607018	81,771,877–82,459,616	GO:0007165 signal transduction<br>GO:0007166 cell surface receptor signaling pathway<br>GO:0007186 G protein-coupled receptor signaling pathway<br>GO:0007189 adenylate cyclase-activating G protein-coupled receptor signaling pathway<br>GO:0007420 brain development

39	*TTLL7*	Tubulin Tyrosine Ligase Like 7	79739	618813	84,330,707–84,464,815	GO:0000226 microtubule cytoskeleton organization<br>GO:0007399 nervous system development<br>GO:0018095 protein polyglutamylation<br>GO:0030154 cell differentiation<br>GO:0036211 protein modification process
40	*PRKACB*	Protein Kinase CAMP-Activated Catalytic Subunit Beta	5567	176892	84,543,762–84,704,181	GO:0001843 neural tube closure<br>GO:0003091 renal water homeostasis<br>GO:0006468 protein phosphorylation<br>GO:0007165 signal transduction<br>GO:0007188 adenylate cyclase-modulating G protein-coupled receptor signaling pathway
41	*SAMD13*	Sterile Alpha Motif Domain Containing 13	148418	–	84,764,221–84,816,481	None

Supplementary Table 2. Deleted protein-coding genes in previously reported 1p31.1 deletion cases with genomic coordinates overlapping with our patient.

Study	Deletion region	Deletion length (Mb)	Number of deleted genes	Protein-coding genes (GRCh37/hg19) [24]
Our case	Chr1: 70,438,241–84,822,837	14.385	41	***LRRC7, LRRC40, SRSF11, ANKRD13C, CTH, PTGER3, ZRANB2, NEGR1, LRRIQ3, FPGT, FPGT-TNNI3K, TNNI3K, LRRC53, ERICH3, CRYZ, TYW3, LHX8, SLC44A5, ACADM, RABGGTB, MSH4, ASB17, ST6GALNAC3, ST6GALNAC5, PIGK, AK5, ZZZ3, USP33, MIGA1, NEXN, FUBP1, DNAJB4, GIPC2, PTGFR, IFI44L, IFI44, ADGRL4, ADGRL2, TTLL7, PRKACB, SAMD13***
Maegawa et al, 2008 [14]	Chr1: 74,413,000-89,929,000	15.46	71	***LRRIQ3, FPGT, FPGT-TNNI3K, TNNI3K, LRRC53, ERICH3, CRYZ, TYW3, LHX8, SLC44A5, ACADM, RABGGTB, MSH4, ASB17, ST6GALNAC3, ST6GALNAC5, PIGK, AK5, ZZZ3, USP33, MIGA1, NEXN, FUBP1, DNAJB4, GIPC2, PTGFR, IFI44L, IFI44, ADGRL4, ADGRL2, TTLL7, PRKACB, SAMD13,*** *DNASE2B, RPF1, GNG5, SPATA1, CTBS, SSX2IP, LPAR3, MCOLN2, MCOLN3, DNAI3, SYDE2, C1orf52, BCL10, DDAH1, CCN1, ZNHIT6, COL24A1, ODF2L, CLCA2, CLCA1, CLCA4, CLCA3P, SH3GLB1, SELENOF, HS2ST1, LMO4, PKN2, GTF2B, KYAT3, RBMXL1, GBP3, GBP1, GBP2, GBP7, GBP4, GBP5, GBP6, GBP1P1*
Callier et al, 2008 [15]	Chr1: 73,900,000-89,500,000	15.6	65	***FPGT, FPGT-TNNI3K, TNNI3K, LRRC53, ERICH3, CRYZ, TYW3, LHX8, SLC44A5, ACADM, RABGGTB, MSH4, ASB17, ST6GALNAC3, ST6GALNAC5, PIGK, AK5, ZZZ3, USP33, MIGA1, NEXN, FUBP1, DNAJB4, GIPC2, PTGFR, IFI44L, IFI44, ADGRL4, ADGRL2, TTLL7, PRKACB, SAMD13,*** *DNASE2B, RPF1, GNG5, SPATA1, CTBS, SSX2IP, LPAR3, MCOLN2, MCOLN3, DNAI3,*

				SYDE2, C1orf52, BCL10, DDAH1, CCN1, ZNHIT6, COL24A1, ODF2L, CLCA2, CLCA1, CLCA4, CLCA3P, SH3GLB1, SELENOF, HS2ST1, LMO4, PKN2, GTF2B, KYAT3, RBMXL1, GBP3, GBP1, GBP2
Chen et al, 2011 [11]	Chr1: 55,500,291–77,711,982	22.2	84	*PCSK9, USP24, PLPP3, PRKAA2, FYB2, C8A, C8B, DAB1, OMA1, TACSTD2, MYSM1, JUN, FGGY, FGGY, HOOK1, CYP2J2, C1orf87, NFIA, TM2D1, PATJ, L1TD1, KANK4, USP1, DOCK7, ANGPTL3, ATG4C, FOXD3, U7, ALG6, ITGB3BP, EFCAB7, DLEU2L, PGM1, ROR1, UBE2U, CACHD1, RAVER2, JAK1, AK4, DNAJC6, LEPROT, LEPR, PDE4B, SGIP1, DYNLT5, INSL5, DNAI4, MIER1, SLC35D1, C1orf141, IL23R, IL12RB2, SERBP1, GADD45A, GNG12, DIRAS3, WLS, RPE65, DEPDC1,* ***LRRC7, LRRC40, SRSF11, ANKRD13C, CTH, PTGER3, ZRANB2, NEGR1, LRRIQ3, FPGT, FPGT-TNNI3K, TNNI3K, LRRC53, ERICH3, CRYZ, TYW3, LHX8, SLC44A5, ACADM, RABGGTB, MSH4, ASB17, ST6GALNAC3, ST6GALNAC5, PIGK***
Yildirim et al, 2014 [16]	Chr1: 75,247,714–75,520,877	0.273	0	None
Genovese et al, 2015 [3]	Chr1: 71,868,625–72,748,533	0.88	1	***NEGR1***
Tassano et al, 2015 [2]	Chr 1: 67,510,407–74,041,202	6	19	*SLC35D1, C1orf141, IL23R, IL12RB2, SERBP1, GADD45A, GNG12, DIRAS3, WLS, RPE65, DEPDC1,* ***LRRC7, LRRC40, SRSF11, ANKRD13C, CTH, PTGER3, ZRANB2, NEGR1***
Rivera-Pedroza et al, 2016 [12]	Chr1: 63,871,758–82,484,133	18.6	69	*ALG6, ITGB3BP, EFCAB7, DLEU2L, PGM1, ROR1, UBE2U, CACHD1, RAVER2, JAK1, AK4, DNAJC6, LEPROT, LEPR, PDE4B, SGIP1, DYNLT5, INSL5, DNAI4, MIER1, SLC35D1, C1orf141, IL23R, IL12RB2, SERBP1, GADD45A, GNG12, DIRAS3, WLS, RPE65, DEPDC1,* ***LRRC7, LRRC40, SRSF11, ANKRD13C, CTH, PTGER3, ZRANB2, NEGR1, LRRIQ3, FPGT, FPGT-TNNI3K, TNNI3K, LRRC53, ERICH3, CRYZ, TYW3, LHX8, SLC44A5, ACADM, RABGGTB, MSH4, ASB17, ST6GALNAC3, ST6GALNAC5, PIGK, AK5, ZZZ3, USP33, MIGA1, NEXN, FUBP1, DNAJB4, GIPC2, PTGFR, IFI44L, IFI44, ADGRL4, ADGRL2***
Biswal et al, 2021 [1]	Chr1: 71,541,998–77,529,328	5.99	18	***ZRANB2, NEGR1, LRRIQ3, FPGT, FPGT-TNNI3K, TNNI3K, LRRC53, ERICH3, CRYZ, TYW3, LHX8, SLC44A5, ACADM, RABGGTB, MSH4, ASB17, ST6GALNAC3, ST6GALNAC5***
Serra et al, 2022 [13]	Chr1: 67,721,572–88,415,438	20.7	76	*IL23R, IL12RB2, SERBP1, GADD45A, GNG12, DIRAS3, WLS, RPE65, DEPDC1,* ***LRRC7, LRRC40, SRSF11, ANKRD13C, CTH, PTGER3, ZRANB2, NEGR1, LRRIQ3, FPGT, FPGT-TNNI3K, TNNI3K, LRRC53, ERICH3, CRYZ, TYW3, LHX8, SLC44A5, ACADM, RABGGTB, MSH4, ASB17, ST6GALNAC3, ST6GALNAC5, PIGK, AK5, ZZZ3, USP33, MIGA1, NEXN, FUBP1, DNAJB4, GIPC2, PTGFR, IFI44L, IFI44, ADGRL4, ADGRL2, TTLL7, PRKACB, SAMD13,*** *DNASE2B, RPF1, GNG5, SPATA1, CTBS, SSX2IP, LPAR3, MCOLN2, MCOLN3, DNAI3, SYDE2, C1orf52, BCL10, DDAH1, CCN1, ZNHIT6, COL24A1, ODF2L, CLCA2, CLCA1, CLCA4, CLCA3P, SH3GLB1, SELENOF, HS2ST1, LMO4*

* Overlapping deleted genes with the present case are in bold
